# Global Burden of *Trichostrongylus* Infections in Humans: A Systematic Review and Meta-Analysis

**DOI:** 10.3390/medicina62020408

**Published:** 2026-02-20

**Authors:** Jurairat Jongthawin, Kinley Wangdi, Aongart Mahittikorn, Frederick Ramirez Masangkay, Manas Kotepui

**Affiliations:** 1 Faculty of Medicine, Mahasarakham University, Maha Sarakham 44000, Thailand; jurairat.j@msu.ac.th; 2 HEAL Global Research Center, Health Research Institute, Faculty of Health, University of Canberra, Bruce ACT 2617, Australia; kinley.wangdi@canberra.edu.au; 3 Department of Protozoology, Faculty of Tropical Medicine, Mahidol University, Bangkok 10400, Thailand; aongart.mah@mahidol.ac.th; 4 Department of Medical Technology, Faculty of Pharmacy, University of Santo Tomas, Manila 1008, Philippines; frmasangkay@ust.edu.ph; 5 Research Center for the Natural and Applied Sciences, University of Santo Tomas, Manila 1008, Philippines; 6 Medical Technology Program, Faculty of Science, Nakhon Phanom University, Nakhon Phanom 48000, Thailand

**Keywords:** *Trichostrongylus*, prevalence, zoonosis, systematic review, meta-analysis, global, burden

## Abstract

*Trichostrongylus* species are zoonotic gastrointestinal nematodes that occasionally infect humans, particularly in rural areas with close contact to livestock. However, the global prevalence of human trichostrongylosis remains uncertain. This systematic review and meta-analysis aimed to synthesize available prevalence data and describe regional and methodological differences in reported infections. Studies published between 2000 and 2025 reporting the prevalence of *Trichostrongylus* infections in humans (primarily *T. colubriformis*, *T. axei*, and *T. orientalis*) were searched in six databases (EMBASE, Ovid, PubMed, Scopus, Nursing & Allied Health Premium, and Web of Science) and Google Scholar. Pooled prevalence was estimated using a random-effects model. Subgroup analyses were conducted to assess prevalence by continent, country, population group, and diagnostic method. Thirty-seven studies from 14 countries, comprising 111,408 participants, were included. Most studies were conducted in Asia (23, 62.2%), particularly in Iran (12, 32.4%), and in Africa (12, 32.4%), mainly in Nigeria (5, 13.5%). The global pooled prevalence of *Trichostrongylus* infection was 1.2%. Prevalence was highest in Africa (1.7%), followed by South America (1.2%), Asia (1.0%), and Europe (0.8%). Subgroup analyses revealed substantial heterogeneity in prevalence across study populations, age groups, and detection methods (*p* < 0.05). Available evidence suggests that human *Trichostrongylus* infection remains a localized but persistent zoonotic concern in specific endemic regions, rather than a globally uniform problem. Diagnostic variability, limited regional coverage, and high heterogeneity highlight the need for standardized molecular diagnostics and broader surveillance to accurately define the global epidemiology of trichostrongylosis.

## 1. Introduction

Trichostrongylosis is a parasitic disease caused by *Trichostrongylus* (Looss, 1905), gastrointestinal nematodes that affect human health in limited regions of the world, particularly in rural and tribal communities with poor hygiene, pastoral livelihoods, and limited access to health services. These parasites are also zoonotic, commonly infecting ruminants and occasionally transmitted to humans [1]. Human infection occurs through the ingestion of third-stage larvae (L3) in contaminated food [1]. While most infections are asymptomatic, some can result in clinical manifestations, including growth retardation in children, eosinophilia in adults, diarrhea, anemia, and emaciation [1,2]. The majority of human infections are attributed to *Trichostrongylus axei* (Cobbold, 1879), *Trichostrongylus colubriformis* (Giles, 1892), and *Trichostrongylus orientalis* (Jimbo, 1914) [1]. Early investigations conducted in Iran using morphological identification of adult worms recovered from infected individuals documented human infections with *T. colubriformis*, *T. axei*, and *T. capricola* (Ghadirian, 1974) across several regions of Iran [3,4], suggesting the zoonotic potential and species diversity of *Trichostrongylus* spp. in humans. Reported risk factors for infection include close contact with livestock such as sheep and cattle, which may serve as a source of contaminated meat and dairy products [5,6], as well as being female and aged 41–50 years [7].

There have been few reports on the prevalence of trichostrongylosis worldwide, largely due to the asymptomatic nature of many infections and the low sensitivity of microscopic examination in detecting small numbers of parasite eggs in human feces [8]. In addition, *Trichostrongylus* infections are frequently underreported because their eggs resemble those of hookworms. A previous systematic review and meta-analysis of trichostrongylosis in Iran, where the highest number of cases has been reported, estimated a pooled seroprevalence of 10% in the general population and found that prevalence increased with age [9]. However, reliable information on the true global prevalence of trichostrongylosis is lacking, and many cases in human populations may remain undiagnosed. To address this gap, the present systematic review and meta-analysis aimed to estimate the overall prevalence of trichostrongylosis in humans worldwide. Subgroup analyses were conducted to examine variations in prevalence by continent, country, population group, and diagnostic method.

## 2. Methods

### 2.1. Data Collection

The review question addressed the pooled prevalence of *Trichostrongylus* infections among participants in the included studies. The review question was structured according to the Population, Exposure, Comparator, and Outcomes (PECO) framework [10]. P represents participants living in endemic areas; E represents *Trichostrongylus* infections; C is none; and O is the pooled prevalence. The context of this review was global.

The searches were conducted in six research databases: EMBASE, Ovid, PubMed, Scopus, Nursing & Allied Health Premium, and Web of Science. The search strategy used the terms ‘*Trichostrongylus*’ and ‘human,’ combined with synonyms and Boolean operators as follows: (*Trichostrongylus* OR *Trichostrongyloidea* OR “*Trichostrongylus colubriformis*” OR “*Trichostrongylus orientalis*” OR “*Trichostrongylus probolurus*”) AND (*human* OR *patients*) (Table S1). An additional search was performed in Google Scholar. Searches were conducted from inception to 27 August 2025. No language restrictions were applied; however, only studies published from 2000 onwards were considered.

Studies were eligible for inclusion if they reported the prevalence of *Trichostrongylus* infections in humans (primarily *T. colubriformis*, *T. axei*, and *T. orientalis*) and reported prevalence data. Only cross-sectional or prevalence studies published in 2000 or later were considered, with no language restrictions. Reviews, abstracts, case reports or case series, outbreak investigations without denominator data, diagnostic test evaluations, errata, and duplicate participant studies were excluded.

All search results from each database were imported into EndNote version 21.0 (Philadelphia, PA, USA). Duplicates were removed, and the remaining studies were screened for relevance by reading titles and abstracts. Non-relevant articles were excluded, and the remaining studies were assessed in full text against the eligibility criteria; those that did not meet the criteria were excluded, with reasons provided. Studies that met the eligibility criteria were included in the final review, and relevant data were extracted into Microsoft Excel 2021 (Microsoft Corporation, Redmond, WA, USA) for further analysis. Study selection and data extraction were performed independently by two review authors (J.J., M.K.), and any disagreements were resolved by discussion until consensus was reached.

The Joanna Briggs Institute (JBI) checklist for prevalence studies was used to assess the risk of bias [11]. Studies with >70% of ‘Yes’ responses were categorized as low risk of bias, those with 50–69% as moderate risk, and those with <50% as high risk [12,13].

The protocol for this systematic review and meta-analysis was registered in PROSPERO (CRD420251117891). This review was reported in accordance with the Preferred Reporting Items for Systematic Reviews and Meta-Analyses (PRISMA) guidelines [14].

### 2.2. Data Synthesis

The pooled prevalence of *Trichostrongylus* infections among participants in the included studies was estimated using a random-effects model with the DerSimonian–Laird method [15]. Heterogeneity was assessed using the *I*^2^ statistic, with values of 25%, 50%, and 75% indicating low, moderate, and high heterogeneity, respectively [16].

Subgroup analyses were performed to examine prevalence according to different characteristics, including continent, country, participant type, age group, and diagnostic method for *Trichostrongylus* species. Sensitivity analysis was carried out by applying a fixed-effects model to compare the pooled prevalence with that obtained from the random-effects model. An influence analysis was performed to identify influential studies (outliers) in the meta-analysis.

Publication bias was assessed using funnel plot inspection and Egger’s regression test [17]. Where asymmetry was observed, the trim-and-fill method was applied to evaluate the potential influence of missing studies on the pooled prevalence estimate [18]. All statistical analyses were conducted using RStudio (Version 2024.04.2 + 764) [19].

## 3. Results

### 3.1. Search Results

A total of 1725 records were initially identified from six research databases (EMBASE, Ovid, PubMed, Scopus, Nursing & Allied Health Premium, and Web of Science). After removing 729 duplicate records, 996 records remained for screening. Of these, 890 records were excluded, primarily because they were unrelated to *Trichostrongylus* (n = 557), were published before 2000 (n = 133), or involved non-human or in vitro samples. At this stage, 106 reports were sought for retrieval, but two could not be obtained, leaving 104 reports for eligibility assessment.

During the full-text assessment, 69 reports were excluded for reasons such as being published before 2000 (n = 43), reporting experimental use of *Trichostrongylus*-positive samples (n = 6), or involving duplicate participant groups (n = 5). Other exclusions included conference abstracts, animal studies, reviews, case reports, duplicate publications, diagnostic test evaluations, and one erratum. Finally, 35 studies [7,20,21,22,23,24,25,26,27,28,29,30,31,32,33,34,35,36,37,38,39,40,41,42,43,44,45,46,47,48,49,50,51,52,53] from the main databases were eligible for inclusion.

**Figure 1:**
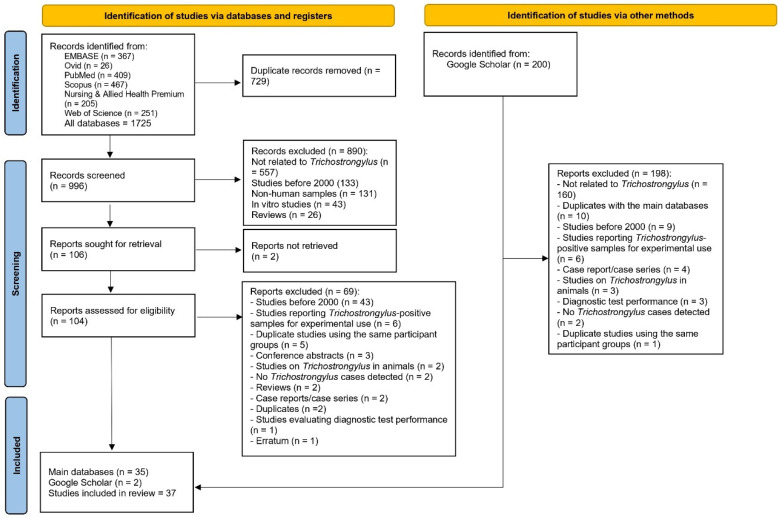
The PRISMA 2020 flow diagram outlines the process of study selection for the review.

In addition, 200 records were identified from Google Scholar. After screening and excluding 198 records, most of which were unrelated or overlapped with the main database search, two additional studies were included. In total, 37 studies [7,20,21,22,23,24,25,26,27,28,29,30,31,32,33,34,35,36,37,38,39,40,41,42,43,44,45,46,47,48,49,50,51,52,53,54,55] met the eligibility criteria and were included in the systematic review (Figure 1).

### 3.2. Characteristics of Included Studies and Risk of Bias

All included studies were cross-sectional and were published between 2005 and 2025 (Table S2). The studies were conducted across four continents, with the majority from Asia (23 studies, 62.2%), primarily in Iran (12 studies, 32.4%) and Lao PDR (5 studies, 13.5%), with additional studies from China, the Republic of Korea, Thailand, and Turkey. The literature from Africa comprised 12 studies (32.4%), mainly from Nigeria (5 studies, 13.5%), with additional contributions from Egypt, Ghana, Mali, South Africa, and Uganda. Only a few studies originated from Europe (Italy, 1 study) and South America (Brazil, 1 study).

Regarding study populations, most were community residents (16 studies, 43.2%), followed by patients with other medical conditions (8 studies, 21.6%) and schoolchildren (5 studies, 13.5%). A smaller number of studies focused on pregnant women, health center attendees, farmers, HIV-infected and uninfected individuals, immigrants, or did not specify the study population. In terms of age groups, the majority included mixed age groups (14 studies, 37.8%), followed by children only (6 studies, 16.2%) and adults only (5 studies, 13.5%). Twelve studies (32.4%) did not report participants’ ages in detail.

For diagnostic methods used to detect *Trichostrongylus*, most studies applied concentration techniques (e.g., formalin-ether concentration, flotation, sedimentation) either alone or in combination with direct smear (32 studies, 83.8%). A smaller number combined concentration methods with agar plate culture (2 studies, 5.4%) or used PCR-based techniques (2 studies, 5.4%). One study relied solely on direct smear microscopy, and another did not specify the diagnostic method (Table 1). Other study-level characteristics are presented in Table S2.

Most of the included studies (33/37; 89.2%) showed a low risk of bias, while four studies (10.8%) had a moderate risk of bias (Table S3). No study was assessed as having a high risk of bias. Therefore, all studies were included in the systematic review and meta-analysis.

### 3.3. Global and Regional Prevalence of Trichostrongylus Infection

**Figure 2:**
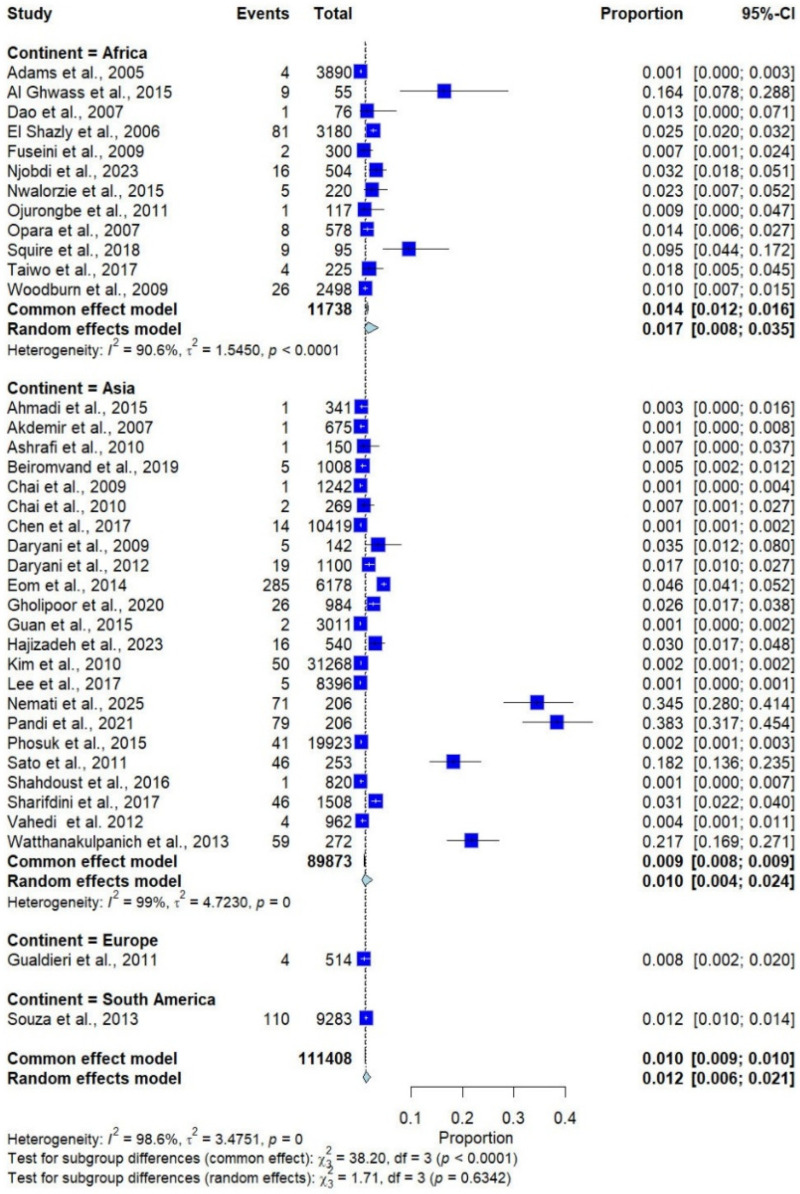
Forest plot of the prevalence of *Trichostrongylus* infections across 37 studies conducted in four continents. Each horizontal line represents the 95% confidence interval (CI) of an individual study, with blue squares indicating the point estimates and their relative weights. The pooled prevalence is shown as a diamond, estimated using both fixed- (common) and random-effects models [7,20,21,22,23,24,25,26,27,28,29,30,31,32,33,34,35,36,37,38,39,40,41,42,43,44,45,46,47,48,49,50,51,52,53,54,55].

Based on a random-effects model, the pooled prevalence of *Trichostrongylus* spp. infections in humans across 14 countries on four continents, with 111,408 participants, were 1.2% (95% confidence interval [CI]: 0.6–2.1%, *I*^2^ = 98.6%; 37 studies, Figure 2). By continent, studies conducted in Africa showed the highest prevalence (1.7%, 95% CI: 0.80–3.50%, *I*^2^: 90.6%, 12 studies), followed by South America (1.2%, 95% CI: 1.00–1.40%, 1 study), Asia (1.0%, 95% CI: 0.40–2.40%, 23 studies), and Europe (0.8%, 95% CI: 0.20–2.00%, 1 study) (Figure 2).

**Figure 3:**
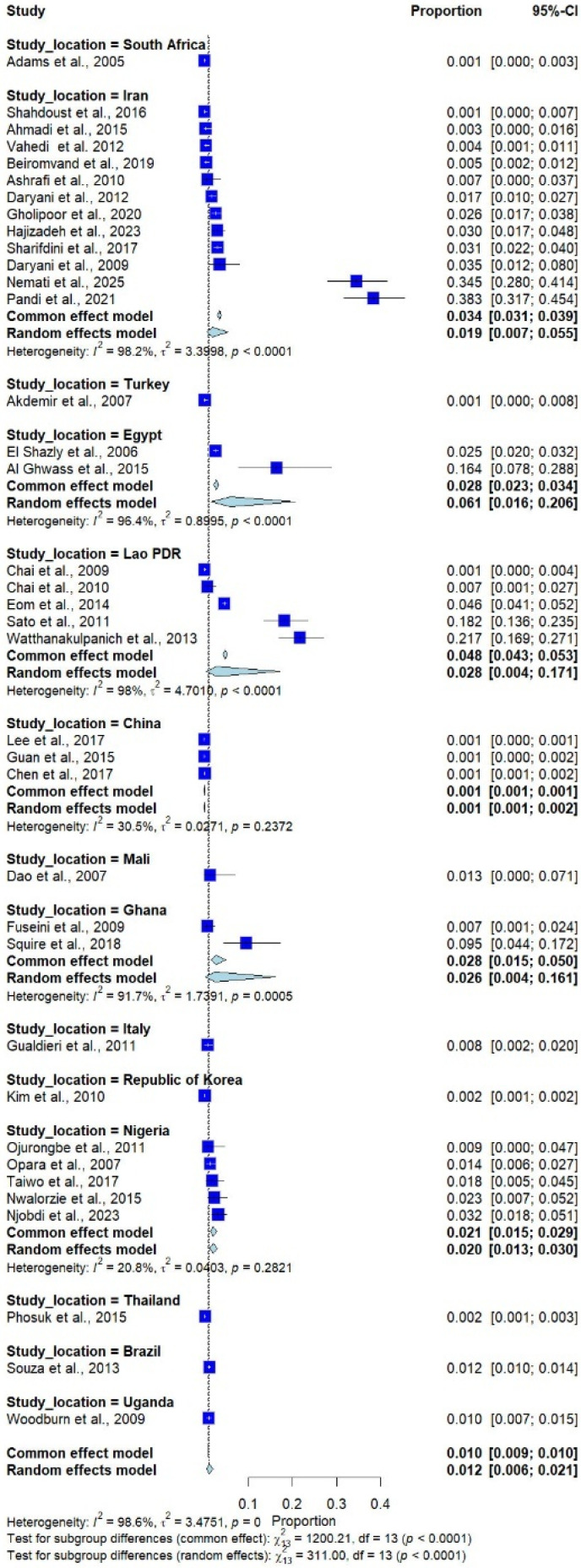
Forest plot of the prevalence of *Trichostrongylus* infections across 37 studies in 14 countries. Each horizontal line represents the 95% confidence interval (CI) of an individual study, with blue squares indicating the point estimates and their relative weights. The pooled prevalence is shown as a diamond, estimated using both fixed- (common) and random-effects models [7,20,21,22,23,24,25,26,27,28,29,30,31,32,33,34,35,36,37,38,39,40,41,42,43,44,45,46,47,48,49,50,51,52,53,54,55].

The subgroup analysis by country, including only those with more than one study, showed that the highest prevalence was observed in Egypt (6.1%; 95% CI: 1.6–20.6%, *I*^2^ = 96.4%, 2 studies), followed by Lao PDR (2.8%; 95% CI: 0.4–17.1%, *I*^2^ = 98.0%, 5 studies), Ghana (2.6%; 95% CI: 0.4–16.1%, *I*^2^ = 91.7%, 2 studies), and Iran (1.9%; 95% CI: 0.7–5.5%, *I*^2^ = 98.2%, 12 studies; Figure 3).

**Figure 4:**
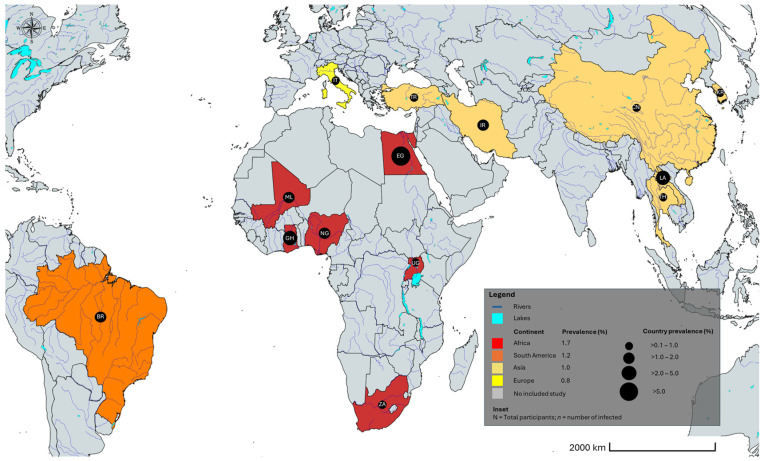
Geographic distribution of *Trichostrongylus* spp. infections worldwide. BR Brazil; CH China; EG Egypt; GH Ghana; IR Iran; IT Italy; KR South Korea; LA Lao People’s Democratic Republic; ML Mali; NG Nigeria; TH Thailand; TR Turkey; UG Uganda; ZA South Africa. The alpha-2 code format was used for country names (https://www.iban.com/country-codes; accessed on 13 September 2025). The map template was sourced from mapchart.net; accessed on 13 September 2025 and annotated by the authors.

The geographical distribution of human *Trichostrongylus* infections worldwide between 2000 and 2025 is shown in Figure 4.

**Figure 5:**
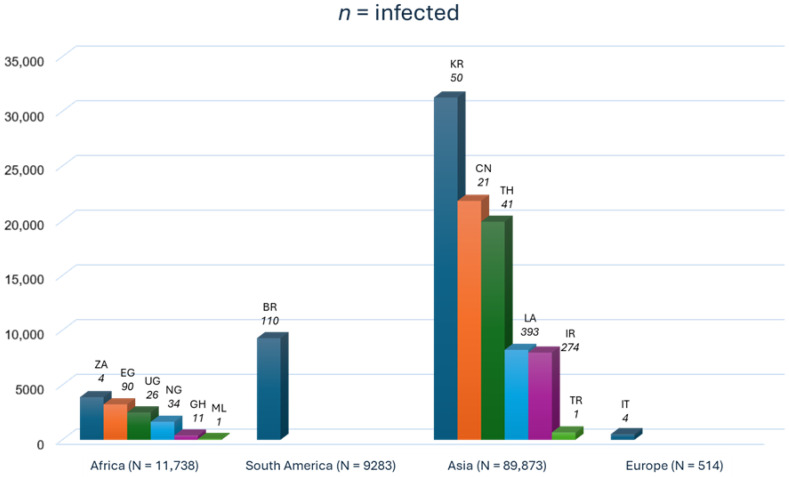
Bar graph of the continental cases of human trichostrongylosis in the included studies. The bar graph shows data on infected participants and the total number of participants per country. BR Brazil; CH China; EG Egypt; GH Ghana; IR Iran; IT Italy; KR South Korea; LA Lao People’s Democratic Republic; ML Mali; NG Nigeria; TH Thailand; TR Turkey; UG Uganda; ZA South Africa. The alpha-2 code format was used for country names (https://www.iban.com/country-codes; accessed on 13 September 2025).

The continental distribution of human trichostrongylosis cases in the included studies is shown in the bar graph (Figure 5).

**Figure 6:**
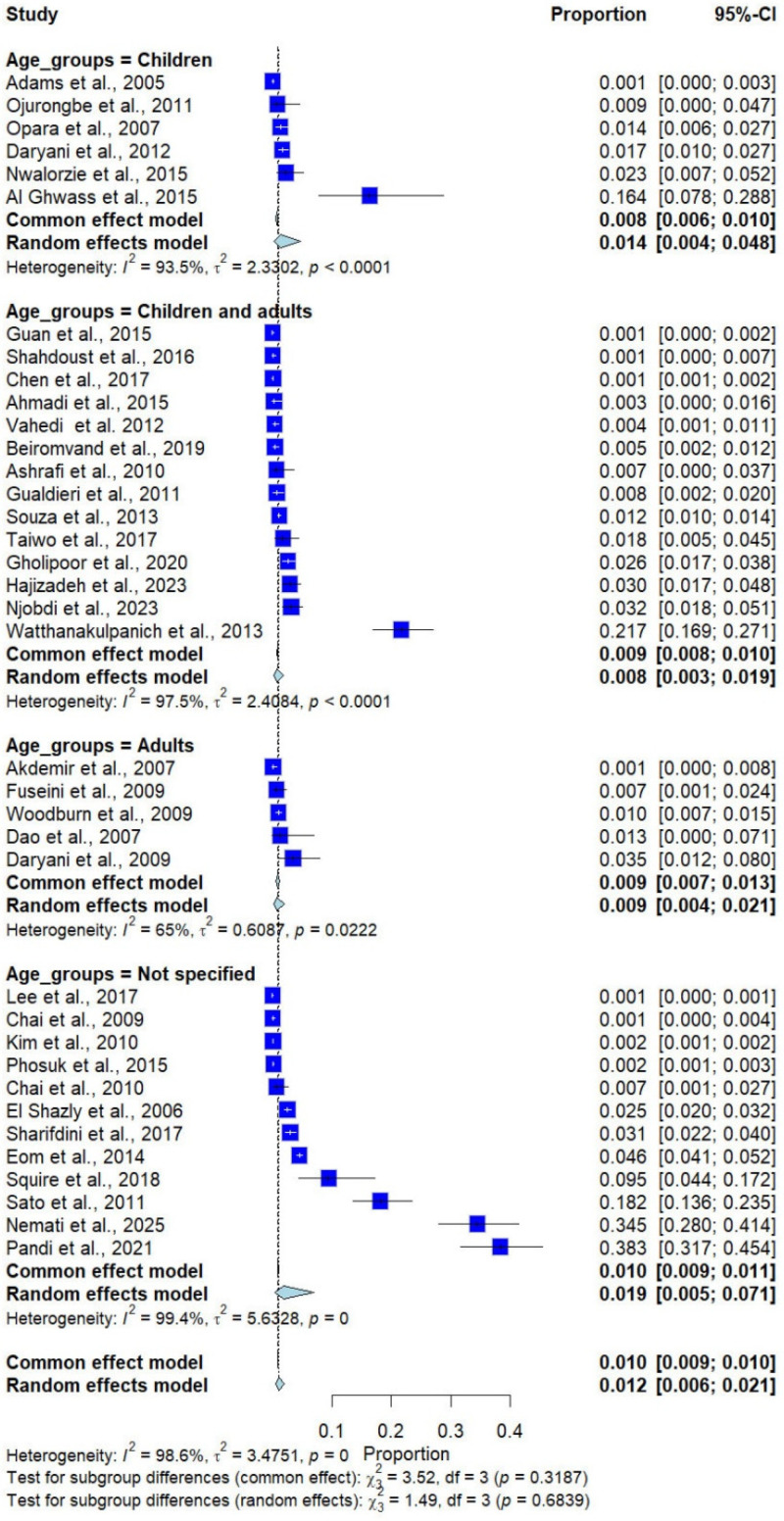
Forest plot of the prevalence of *Trichostrongylus* infections categorized by age groups. Each horizontal line represents the 95% confidence interval (CI) of an individual study, with blue squares indicating the point estimates and their relative weights. The pooled prevalence is shown as a diamond, estimated using both fixed- (common) and random-effects models [7,20,21,22,23,24,25,26,27,28,29,30,31,32,33,34,35,36,37,38,39,40,41,42,43,44,45,46,47,48,49,50,51,52,53,54,55].

Subgroup analysis by age group showed no statistically significant differences (*p* = 0.684). The pooled prevalence of *Trichostrongylus* infections was 1.4% (6 studies) in children, 0.8% (14 studies) in combined children and adults, 0.9% (5 studies) in adults, and 1.9% (12 studies) in unspecified age groups (Figure 6).

**Figure 7:**
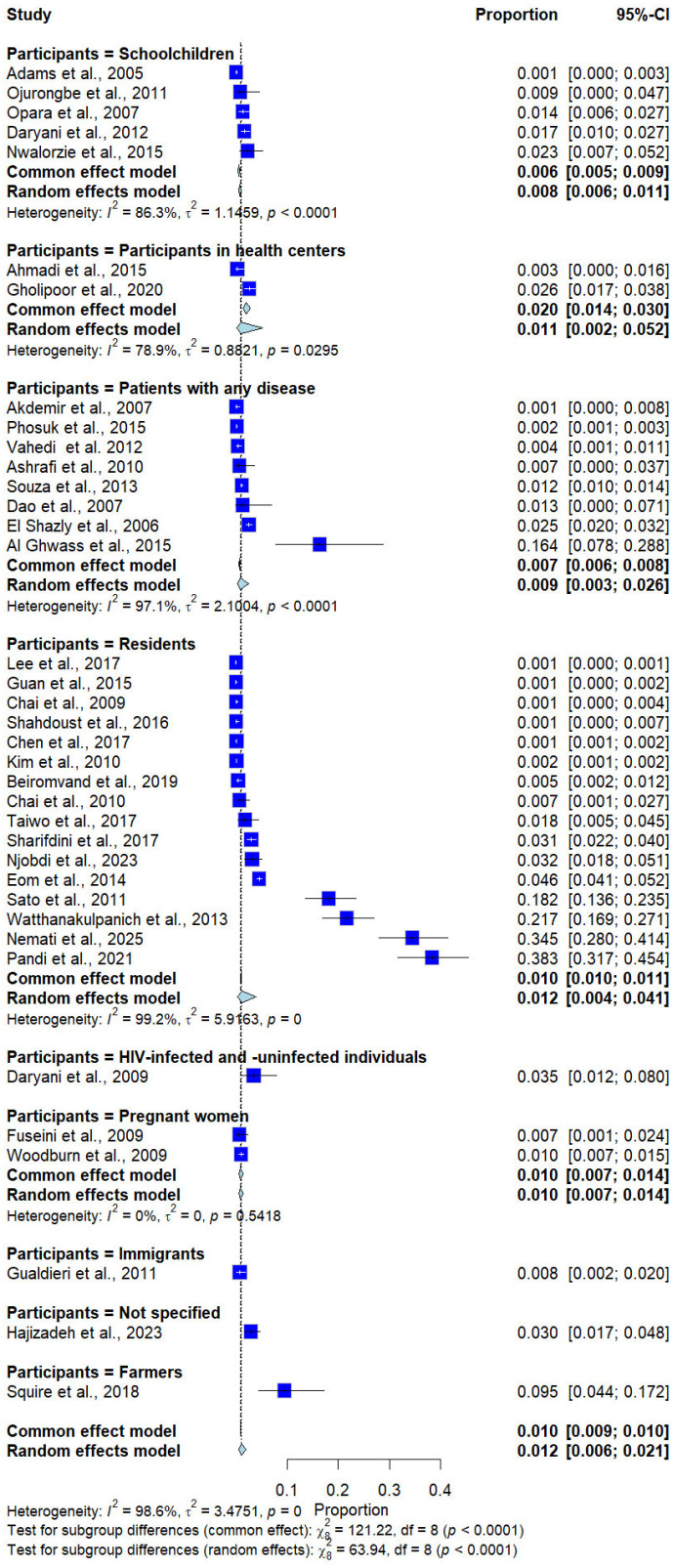
Forest plot of the prevalence of *Trichostrongylus* infections categorized by participant types. Each horizontal line represents the 95% confidence interval (CI) of an individual study, with blue squares indicating the point estimates and their relative weights. The pooled prevalence is shown as a diamond, estimated using both fixed- (common) and random-effects models [7,20,21,22,23,24,25,26,27,28,29,30,31,32,33,34,35,36,37,38,39,40,41,42,43,44,45,46,47,48,49,50,51,52,53,54,55].

Subgroup analysis by participant type revealed significant differences in prevalence (*p* < 0.0001). Higher prevalence estimates were observed in some population groups, such as farmers (9.5%, 1 study) and HIV-infected and uninfected participants (3.5%, 1 study); however, these estimates were derived from a small number of studies and a heterogeneous population (Figure 7).

**Figure 8:**
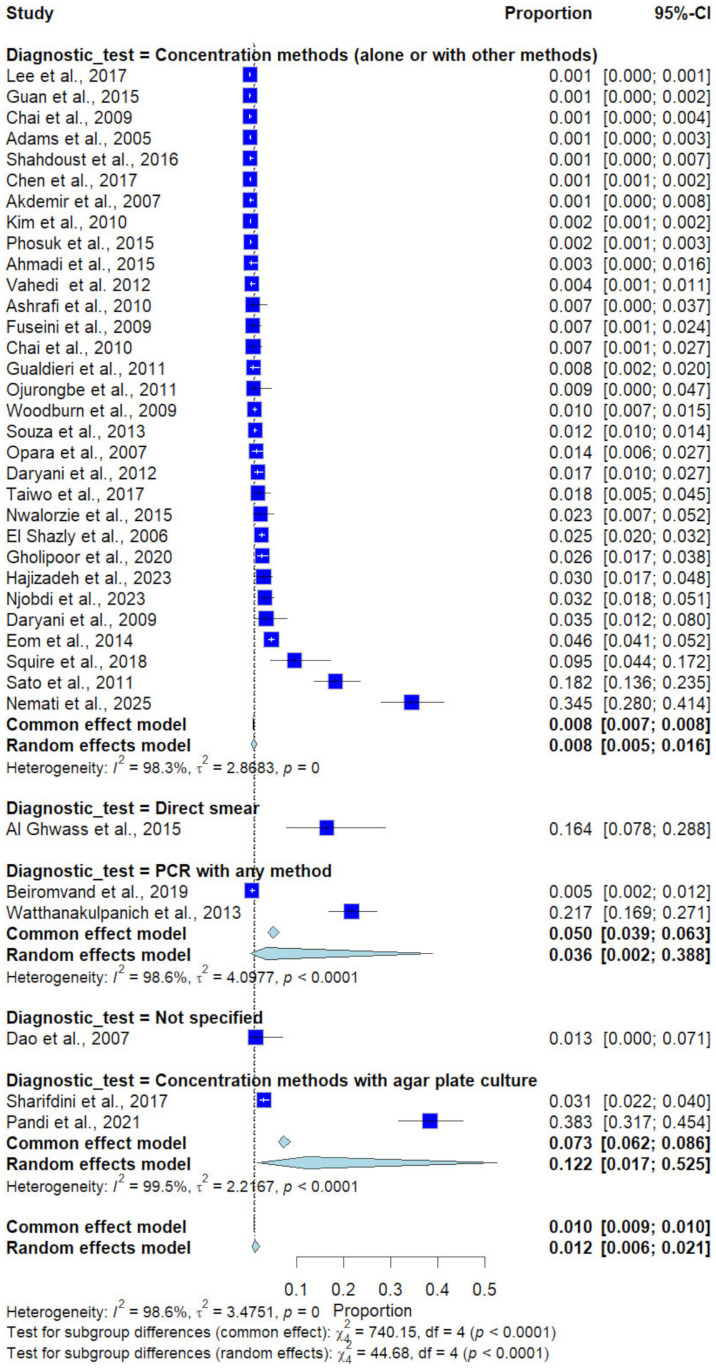
Forest plot of the prevalence of *Trichostrongylus* infections categorized by diagnostic methods. Each horizontal line represents the 95% confidence interval (CI) of an individual study, with blue squares indicating the point estimates and their relative weights. The pooled prevalence is shown as a diamond, estimated using both fixed- (common) and random-effects models [7,20,21,22,23,24,25,26,27,28,29,30,31,32,33,34,35,36,37,38,39,40,41,42,43,44,45,46,47,48,49,50,51,52,53,54,55].

Subgroup analysis by diagnostic method demonstrated significant differences (*p* < 0.0001). Studies using concentration methods alone or in combination reported a prevalence of 0.8% (31 studies), whereas studies using direct smear reported a prevalence of 16.4% (1 study), and those using concentration methods with agar plate culture reported a prevalence of 12.2% (2 studies). PCR-based methods showed intermediate prevalence (3.6%, 2 studies), and studies with unspecified diagnostic methods reported 1.3% (1 study) (Figure 8).

### 3.4. Sensitivity Analysis and Publication Bias

In the meta-analysis of *Trichostrongylus* infections, the random-effects model estimated a pooled prevalence of 1.12% (95% CI: 0.6–2.1%), while the common-effect model yielded a slightly lower estimate of 1.0% (95% CI: 0.9–1.0%) (Figure 2). The leave-one-out (influential) analysis showed that the overall pooled prevalence estimate of *Trichostrongylus* infection remained stable, ranging between about 0.74% and 1.26% when individual studies were excluded. No single study had a disproportionate effect on the overall results (Table S4).

**Figure 9:**
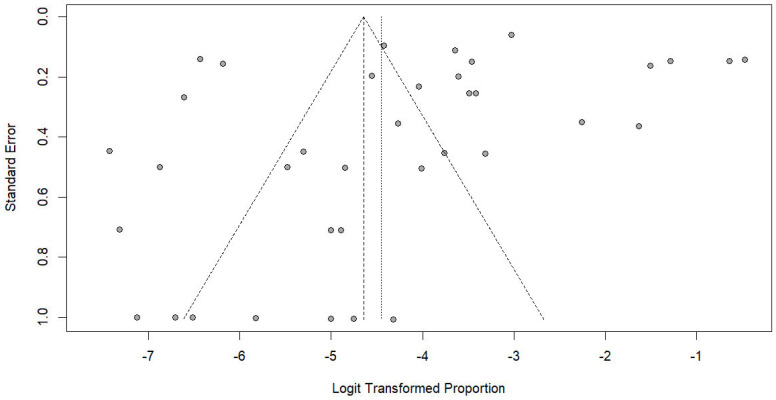
Funnel plot assessing publication bias in studies reporting the prevalence of *Trichostrongylus* infections. Each point represents a study plotted by its standard error against the logit-transformed proportion. The vertical lines indicate the pooled estimate and pseudo 95% confidence limits.

The funnel plot showed minor asymmetry (Figure 9), but Egger’s regression test did not indicate significant publication bias (*p* = 0.136), suggesting that the pooled estimates were unlikely to be substantially influenced by selective reporting or small-study effects. The trim-and-fill method indicated that after 14 studies were added to the meta-analysis, the estimated prevalence of *Trichostrongylus* infections in humans was 4.29% (95% CI: 2.13; 8.48, *I*^2^: 98.8%, 51 studies).

Overall, reported human *Trichostrongylus* infections were confined to a limited number of countries, predominantly in Asia and Africa, with sporadic reports from Europe and South America. Most prevalence estimates were low (<2%), and cases were concentrated in rural or livestock-associated settings. Large geographic regions, including North America and most of Europe and South America, lacked population-based prevalence data.

## 4. Discussion

The present systematic review and meta-analysis collated and updated evidence on the prevalence of human *Trichostrongylus* infections based on studies published between 2000 and 2025. Across 37 studies including more than 111,000 participants from 14 countries and four continents, the pooled prevalence was 1.2%, indicating that human *Trichostrongylus* infection is uncommon and largely confined to specific endemic settings. Although some geographic variation was observed, with slightly higher pooled prevalence in Africa (1.7%) than in South America (1.2%), Asia (1.0%), and Europe (0.8%), these differences were small and should be interpreted cautiously, considering substantial heterogeneity and uneven geographic representation. Overall, despite its zoonotic nature and widespread distribution, *Trichostrongylus* infection remains a minor helminthiasis in humans compared with other soil-transmitted helminths.

The differences in pooled prevalence across continents may be explained by ecological, occupational, and cultural factors influencing exposure. Ecologically, soil surface temperature and the timing and amount of rainfall affect the development and survival of infective larvae of *Trichostrongylus* spp. [56,57]. Parasite eggs in feces do not develop into infective larvae during the winter season [57]. Therefore, regions with long winters, such as Europe, may have a lower risk of transmission for *Trichostrongylus* infections than continents with hotter, rainier seasons, such as Africa, South America, and Asia. Moreover, agricultural and pastoral communities in Africa and Asia [58] may be associated with close contact with livestock, facilitating zoonotic transmission. The dataset is geographically unbalanced, as most studies originated in Iran and Nigeria, while several regions, including North America, most of Europe, and large parts of South America, remain underrepresented. Therefore, the results should not be interpreted as a fully representative estimate of the global prevalence, but rather as a synthesis of available evidence from currently studied regions.

In some areas, foodborne transmission through the consumption of raw or undercooked vegetables may increase the risk of infection. Previous studies have documented the presence of *Trichostrongylus* spp. in various food sources, particularly leafy vegetables [59], retail and farm produce [60], parsley and radish [61], spinach [62], unwashed vegetables [63], raw vegetables [64,65], and other plants such as white jute, pumpkin, and quill grass [66]. Irrigation with contaminated water has also been associated with an increased risk of infection. Previous studies have documented the presence of *Trichostrongylus* species in various environmental sources, including rivers in flood-prone areas [67], public swimming pools, and drinking water [68], soil samples [69,70,71,72,73], treated wastewater samples [74], wastewater samples [75], untreated entry wastewater [76], and household water supplies [77]. One study further suggested that *Trichostrongylus* larvae can survive thermal treatment [78], indicating that the use of sewage sludge and agricultural residues following such processes may pose contamination risks for both humans and animals. In contrast, lower prevalence in Europe may be explained by better sanitation, improved food safety measures, and limited exposure to contaminated environments. Moreover, wildlife consumed as meat, small ruminants such as sheep and goats, and slaughtered cattle have all been reported as potential sources of *Trichostrongylus* infection in humans [79,80,81]. For example, monkeys living in densely urbanized areas may also contribute to transmission [82,83,84].

Subgroup analyses of the studied populations showed a higher prevalence among farmers (9.5%) and among both HIV-infected and uninfected individuals (3.5%), although only a few studies were included in these subgroups. Farmers may be at elevated risk due to daily exposure to contaminated soil, livestock, and animal waste, while the higher prevalence among HIV-related participants is likely due to reduced immune responses to infection. In comparison, schoolchildren, residents, and pregnant women showed relatively lower prevalence (<1.2%), suggesting that community transmission may be sporadic rather than widespread. These findings also suggest that while most infections are asymptomatic in the general healthy population, clinical manifestations can occur among high-risk groups. In addition, subgroup analysis by age group showed a higher prevalence in children (1.4%) than in adults (0.9%) and mixed-age groups (0.8%). These results suggest that children may be more susceptible to *Trichostrongylus* infection due to factors such as limited personal hygiene (e.g., playing in soil contaminated with animal feces during outdoor activities) or immature immune systems.

The present meta-analysis assessed *Trichostrongylus* infections at the genus level because most included studies did not report species-specific data. Among those that did, *T. colubriformis*, *T. axei*, and *T. orientalis* were the most frequently identified species infecting humans, with occasional reports of *T. probolurus* and other species detected by molecular methods. However, due to insufficient species-level information, quantitative comparisons were not possible. Another important consideration is the diagnostic challenge posed by this parasite: *Trichostrongylus* eggs are morphologically similar to those of hookworms, leading to misdiagnosis and inaccurate prevalence estimates. There was a significant difference in infection prevalence depending on the diagnostic method used. Subgroup analysis showed that studies employing direct smear or agar plate culture reported markedly higher estimates compared with those using concentration methods. This discrepancy may be explained by differences in methodological sensitivity and specificity; direct smear, while less commonly used, may misclassify hookworm eggs as *Trichostrongylus*, thereby inflating prevalence, whereas concentration methods, though widely applied, may underestimate infection due to low egg burdens and intermittent shedding. Subgroup analysis also demonstrated the relatively higher prevalence detected by PCR, underscoring the value of molecular approaches in improving diagnostic accuracy. Several molecular methods have been developed for detecting *Trichostrongylus* spp. For example, PCR with high-resolution melting (PCR-HRM) targeting the ITS-2 rDNA region has been shown to be a rapid and low-cost approach, with results comparable to other molecular techniques [85]. Multiplex RE-PCR using the restriction enzymes *HinfI*, *DraI*, and *MseI* has also proven useful for discriminating among *Trichostrongylus* species [86]. In addition, real-time PCR with primers and probes specific to the 5.8S and ITS-2 regions of *Trichostrongylus* rDNA has been suggested as a fast and specific method for detecting infections in fecal specimens and distinguishing zoonotic species such as *T. colubriformis* and *T. axei* [87]. Furthermore, PCR amplification of the ITS-1 sequence can differentiate *Trichostrongylus* eggs from hookworm eggs, which is particularly useful for epidemiological studies in co-endemic regions such as the Lao PDR [88]. While these molecular assays provide greater sensitivity and specificity than traditional methods, they remain underutilized in endemic regions due to cost and technical constraints. Although the analysis showed that prevalence estimates were generally higher in studies using molecular or culture-based methods than in those using conventional microscopy, reflecting the greater accuracy of these techniques, most included studies relied on microscopic identification without molecular confirmation. Therefore, some degree of diagnostic misclassification cannot be ruled out.

The findings of this study provide several public health implications. First, although the global prevalence of *Trichostrongylus* infection appears low, underdiagnosis and misclassification likely obscure its true burden. Routine stool examinations often fail to distinguish *Trichostrongylus* eggs from hookworm eggs, leading to misdiagnosis and inappropriate treatment. Second, the zoonotic nature of this parasite emphasizes the need for integrated One Health surveillance that connects human, animal, and environmental health. Third, targeted preventive measures, such as promoting safe farming practices, improving vegetable hygiene, and strengthening diagnostic capacity, could reduce infection risk in high-exposure groups. This study also has limitations. First, the substantial heterogeneity (*I*^2^ > 98%) likely reflects the influence of unmeasured environmental and socio-economic factors, including climate, livestock density, sanitation, and agricultural practices, which could not be quantitatively assessed due to limited data. As a result, the analysis may not fully capture the contextual drivers of infection, especially those explaining geographical and temporal differences. Second, groups such as pregnant women, migrants, farmers, and community residents represent fundamentally different population definitions and exposure contexts and are not directly comparable. Consequently, subgroup prevalence estimates should be interpreted as descriptive and exploratory, and not as definitive indicators of relative risk between population groups. The lack of individual participant data and inconsistent reporting of covariates across studies precluded multivariate analyses accounting for overlapping participant characteristics (e.g., pregnancy status, migration, occupation, and environmental exposure). Therefore, subgroup findings should be interpreted as exploratory and descriptive rather than as independent predictors of infection risk. Third, the geographic distribution presented in this review reflects only countries for which population-based prevalence data were available. Also, case-based reports were therefore excluded because they lacked prevalence estimates. As a result, the mapped distribution should not be interpreted as a comprehensive representation of the global presence of *Trichostrongylus*, but rather as a summary of locations with eligible prevalence studies. Fourth, because most included studies used low-sensitivity microscopic methods, the pooled global prevalence should be interpreted as a conservative estimate rather than a true reflection of the global burden. The apparent low prevalence likely reflects diagnostic heterogeneity and under-detection. Despite these limitations, this study provides the first systematic synthesis of human *Trichostrongylus* prevalence across multiple continents, summarizing available evidence from 25 years of research. The findings highlight the continuing presence of this relatively uncommon zoonotic infection and the diagnostic and surveillance gaps that hinder accurate global assessment. Rather than presenting a definitive global estimate, this analysis establishes a foundation for future regionally representative studies that incorporate standardized molecular diagnostics and environmental data.

## 5. Conclusions

This systematic review and meta-analysis provide the most comprehensive synthesis to date of reported human *Trichostrongylus* infections between 2000 and 2025, based on available but regionally limited evidence. Although infections were detected across four continents, the majority of studies originated from Iran and Nigeria, resulting in uneven geographical representation and limiting the generalizability of the pooled estimate. Some population groups with increased environmental or occupational exposure, such as farmers, showed higher prevalence estimates in individual studies. Given the dominance of low-sensitivity microscopic diagnostic methods and the absence of data from large parts of the world, the reported prevalence likely underestimates the true burden. Rather than a definitive estimate of global prevalence, these results should be viewed as a baseline summary of currently available data. Future research should prioritize expanding surveillance to underrepresented regions, incorporating environmental and socio-economic determinants, and adopting standardized molecular diagnostics and species-level reporting to better characterize the distribution and zoonotic potential of *Trichostrongylus* infections. Overall, the findings provide updated evidence on human trichostrongylosis and may help inform future research on its epidemiology, pathogenicity, and zoonotic potential.

## Figures and Tables

**Table 1:** Summary characteristics of included studies (n = 37).

Publication Year	n.	%
2000–2009	9	24.3
2010–2019	23	62.2
2020–2025	5	13.5
Regions (Continents and Countries)		
Africa	12	32.4
Nigeria	5	13.5
Ghana	2	5.4
Uganda	1	2.7
Egypt	2	5.4
South Africa	1	2.7
Mali	1	2.7
Asia	23	62.2
Iran	12	32.4
China	3	8.1
Lao PDR	5	13.5
Republic of Korea	1	2.7
Thailand	1	2.7
Turkey	1	2.7
Europe	1	2.7
Italy	1	2.7
South America	1	2.7
Brazil	1	2.7
Participants		
Residents	16	43.2
Farmers	1	2.7
Immigrants	1	2.7
Schoolchildren	5	13.5
Pregnant women	2	5.4
Participants in health centers	2	5.4
Patients with any disease	8	21.6
HIV-infected and -uninfected individuals	1	2.7
Not specified	1	2.7
Age groups		
Children	6	16.2
Adults	5	13.5
Children and adults	14	37.8
Not specified	12	32.4
Detection method		
Concentration methods (alone or with other methods)	31	83.8
Concentration methods with agar plate culture	2	5.4
PCR with any method	2	5.4
Direct smear	1	2.7
Not specified	1	2.7

## Data Availability

All data relating to the present study are available in this manuscript, Tables S1–S4.

## Supplementary Materials

The following supporting information can be downloaded at: https://www.mdpi.com/article/10.3390/medicina62020408/s1, Table S1: Search terms; Table S2: Details of included studies; Table S3: Methodological quality of the include studies; Table S4: The leave-one-out (influential) analysis.
