# Supplementary material for: Global Burden of Trichostrongylus Infections in Humans: A Systematic Review and Meta-Analysis
Source: Medicina (Kaunas). 2026 Feb 20;62(2):408. doi: 10.3390/medicina62020408 (PMC12942499; doi:10.3390/medicina62020408)
Supplement: Supplementary file 1 [file medicina-62-00408-s001.zip › Table S4. The leave-one-out (influential) analysis.pdf]

Global prevalence of *Trichostrongylus* infections in humans: A systematic review and meta-analysis

Jurairat Jongthawin[1], Kinley Wangdi[2], Frederick Ramirez Masangkay[3,4], Manas Kotepui[5,*]

[1]Faculty of Medicine, Mahasarakham University, Maha Sarakham 44000, Thailand

[2]HEAL Global Research Center, Health Research Institute, Faculty of Health, University of Canberra, Bruce, ACT 2617, Australia

[3]Department of Medical Technology, Faculty of Pharmacy, University of Santo Tomas, Manila 1008, Philippines

[4]Research Center for the Natural and Applied Sciences, University of Santo Tomas, Manila 1008, Philippines

[5]Medical Technology Program, Faculty of Science, Nakhon Phanom University, Nakhon Phanom 48000, Thailand

*Corresponding author

Jurairat Jongthawin: jurairat.j@msu.ac.th

Kinley Wangdi: Kinley.Wangdi@canberra.edu.au

Frederick Ramirez Masangkay: frmasangkay@ust.edu.ph

Manas Kotepui :manaskote@gmail.com, Tel+ :.66954392469

Table S4. The leave-one-out (influential) analysis

Influential analysis (common effect model)

	proportion	95%-CI	p-value	tau^2	tau	I^2
Omitting Adams et al. [20]	0.0098	[0.0092; 0.0104]		3.3819	1.8390	98.6%
Omitting Ahmadi et al. [21]	0.0095	[0.0090; 0.0101]		3.4965	1.8699	98.6%
Omitting Akdemir et al. [22]	0.0096	[0.0090; 0.0102]		3.4380	1.8542	98.6%
Omitting Al Ghwass et al. [23]	0.0094	[0.0089; 0.0100]		3.3634	1.8340	98.6%
Omitting Ashrafi et al. [24]	0.0095	[0.0090; 0.0101]		3.5398	1.8814	98.6%
Omitting Beiromvand et al. [25]	0.0096	[0.0090; 0.0101]		3.5486	1.8838	98.6%
Omitting Chai et al. [26]	0.0096	[0.0091; 0.0102]		3.3678	1.8352	98.6%
Omitting Chai et al. [27]	0.0095	[0.0090; 0.0101]		3.5567	1.8859	98.6%
Omitting Chen et al. [28]	0.0104	[0.0098; 0.0110]		3.4201	1.8494	98.5%
Omitting Dao et al. [29]	0.0095	[0.0090; 0.0101]		3.5544	1.8853	98.6%
Omitting Daryani et al. [30]	0.0095	[0.0089; 0.0101]		3.5519	1.8847	98.6%
Omitting Daryani et al. [31]	0.0094	[0.0089; 0.0100]		3.5809	1.8923	98.6%
Omitting El Shazly et al. [32]	0.0090	[0.0085; 0.0096]		3.5683	1.8890	98.6%
Omitting Eom et al. [33]	0.0074	[0.0069; 0.0079]		3.5268	1.8780	98.6%
Omitting Fuseini et al. [54]	0.0095	[0.0090; 0.0101]		3.5526	1.8848	98.6%
Omitting Gholipoor et al. [34]	0.0094	[0.0088; 0.0099]		3.5665	1.8885	98.6%
Omitting Gualdieri et al. [35]	0.0095	[0.0090; 0.0101]		3.5676	1.8888	98.6%
Omitting Guan et al. [36]	0.0098	[0.0092; 0.0104]		3.3223	1.8227	98.6%
Omitting Hajizadeh et al. [37]	0.0094	[0.0089; 0.0100]		3.5607	1.8870	98.6%
Omitting Kim et al. [38]	0.0126	[0.0119; 0.0134]		3.4451	1.8561	98.3%
Omitting Lee et al. [39]	0.0102	[0.0096; 0.0109]		3.2883	1.8134	98.6%
Omitting Nemati et al. [40]	0.0089	[0.0084; 0.0095]		3.1355	1.7707	98.4%
Omitting Njobdi et al. [41]	0.0094	[0.0089; 0.0100]		3.5567	1.8859	98.6%

Omitting Nwalorzie et al. [42]	0.0095 [0.0089; 0.0101]	3.5712	1.8898	98.6%
Omitting Ojurongbe et al. [43]	0.0095 [0.0090; 0.0101]	3.5473	1.8834	98.6%
Omitting Opara et al. [55]	0.0095 [0.0089; 0.0101]	3.5814	1.8925	98.6%
Omitting Pandi et al. [44]	0.0088 [0.0083; 0.0094]	3.0932	1.7588	98.3%
Omitting Phosuk et al. [45]	0.0111 [0.0105; 0.0118]	3.4772	1.8647	98.4%
Omitting Sato et al. [46]	0.0091 [0.0086; 0.0097]	3.3217	1.8225	98.5%
Omitting Shahdoust et al. [47]	0.0096 [0.0090; 0.0102]	3.4174	1.8486	98.6%
Omitting Sharifdini et al. [48]	0.0092 [0.0087; 0.0098]	3.5586	1.8864	98.6%
Omitting Souza et al. [49]	0.0093 [0.0087; 0.0099]	3.5852	1.8935	98.5%
Omitting Squire et al. [50]	0.0094 [0.0089; 0.0100]	3.4527	1.8582	98.6%
Omitting Taiwo et al. [51]	0.0095 [0.0089; 0.0101]	3.5756	1.8909	98.6%
Omitting Vahedi et al. [52]	0.0096 [0.0090; 0.0102]	3.5353	1.8802	98.6%
Omitting Watthanakulpanich et al. [7]	0.0090 [0.0085; 0.0096]	3.2782	1.8106	98.5%
Omitting Woodburn et al. [53]	0.0095 [0.0089; 0.0101]	3.5830	1.8929	98.6%
Pooled estimate	0.0095 [0.0090; 0.0101]	3.4751	1.8642	98.6%
