# Supplementary material for: Global Burden of Trichostrongylus Infections in Humans: A Systematic Review and Meta-Analysis
Source: Medicina (Kaunas). 2026 Feb 20;62(2):408. doi: 10.3390/medicina62020408 (PMC12942499; doi:10.3390/medicina62020408)
Supplement: Supplementary file 1 [file medicina-62-00408-s001.zip › Table S1. Search terms.pdf]

Table S1. Search terms

General keywords

(Trichostrongylus OR Trichostrongyloidea OR “Trichostrongylus colubriformis” OR “Trichostrongylus orientalis” OR “Trichostrongylus probolurus”) AND (human OR patients)

PubMed 25 July 2025

No.	Key concept	Search terms	Results
1.	Trichostrongylus	Trichostrongylus[MeSH Terms] OR Trichostrongyloidea[MeSH Terms] OR “Trichostrongylus colubriformis”[MeSH Terms] OR “Trichostrongylus orientalis”[MeSH Terms] OR “Trichostrongylus probolurus”[MeSH Terms] OR Trichostrongylus[Text Word] OR Trichostrongyloidea[Text Word] OR “Trichostrongylus colubriformis” [Text Word] OR “Trichostrongylus orientalis”[Text Word] OR “Trichostrongylus probolurus”[Text Word]	6,938
2.	Human	human[MeSH Terms] OR patients[MeSH Terms] OR human[Text Word] OR patients[Text Word]	24,786,802
3.	1 AND 2	#1 AND #2	409

Embase 25 July 2025

No.	Key concept	Search terms	Results
1.	Trichostrongylus	('trichostrongylus'/exp OR 'trichostrongyloidea'/exp) AND 'trichostrongylus colubriformis'/exp OR 'trichostrongylus orientalis'/exp OR 'trichostrongylus probolurus' OR trichostrongylus:ti,ab,kw,de OR trichostrongyloidea:ti,ab,kw,de OR 'trichostrongylus colubriformis':ti,ab,kw,de OR 'trichostrongylus orientalis':ti,ab,kw,de OR 'trichostrongylus probolurus':ti,ab,kw,de	4,099
2.	Human	'human'/exp OR 'patients'/exp OR human:ti,ab,kw,de OR patients:ti,ab,kw,de	31,855,314
3.	1 AND 2	#1 AND #2	367

Scopus 25 July 2025

No.	Key concept	Search terms	Results
1.	Trichostrongylus	TITLE-ABS-KEY (Trichostrongylus OR Trichostrongyloidea OR "Trichostrongylus colubriformis" OR "Trichostrongylus orientalis" OR "Trichostrongylus probolurus")	5,497

2.	Human	TITLE-ABS-KEY (human OR patients)	30,485,350
3.	1 AND 2	(TITLE-ABS-KEY (Trichostrongylus OR Trichostrongyloidea OR "Trichostrongylus colubriformis" OR "Trichostrongylus orientalis" OR "Trichostrongylus probolurus")) AND (TITLE-ABS-KEY (human OR patients))	467

Ovid (Journal) 25 July 2025

No.	Key concept	Search terms	Results
1.	Trichostrongylus AND Human	(Trichostrongylus OR Trichostrongyloidea OR "Trichostrongylus colubriformis" OR "Trichostrongylus orientalis" OR "Trichostrongylus probolurus") {Including Limited Related Terms}	26

Nursing & Allied Health Premium 25 July 2025

No.	Key concept	Search terms	Results
1.	Trichostrongylus AND Human	(Trichostrongylus OR Trichostrongyloidea OR “Trichostrongylus colubriformis” OR “Trichostrongylus orientalis” OR “Trichostrongylus probolurus”) AND (human OR patients)	205

Web of Science 25 July 2025

No.	Key concept	Search terms	Results
1.	Trichostrongylus AND Human	(Trichostrongylus OR Trichostrongyloidea OR “Trichostrongylus colubriformis” OR “Trichostrongylus orientalis” OR “Trichostrongylus probolurus”) AND (human OR patients) (All Fields)	251

Google Scholar 27 August 2025

No.	Key concept	Search terms	Results
1.	Trichostrongylus AND Human	Trichostrongylus AND Human	18,100 (Screening for the first 200 results)

Google Scholar (n = 200)

160 non-related articles

40 related articles

36 were excluded with reasons;

- Studies before 2000 (n = 9)
- Duplicates with the main databases (n = 10)
- *Trichostrongylus*-positive samples for experiment (n = 6)
- Case report/case series (n = 4)
- Animal samples (n = 3)
- Diagnostic test performance (n = 3)
- No *Trichostrongylus* case (n = 2)
- Studies using the same group of participants (n = 1)

Final included (n = 2)
